# Chimeric Antigen Receptor Immunotherapy for Infectious Diseases: Current Advances and Future Perspectives

**DOI:** 10.3390/pathogens14080774

**Published:** 2025-08-05

**Authors:** Maria Kourti, Paschalis Evangelidis, Emmanuel Roilides, Elias Iosifidis

**Affiliations:** 1 Children & Adolescent Hematology-Oncology Unit, Second Department of Pediatrics, School of Medicine, Aristotle University of Thessaloniki, 54124 Thessaloniki, Greece; 2 Second Propedeutic Department of Internal Medicine, Aristotle University of Thessaloniki, Hippokration General Hospital, 54642 Thessaloniki, Greece; pascevan@auth.gr; 3 Third Department of Pediatrics, Aristotle University of Thessaloniki, Hippokration General Hospital, 54642 Thessaloniki, Greece; roilides@auth.gr (E.R.); iosifidish@gmail.com (E.I.)

**Keywords:** chimeric antigen receptor, human immunodeficiency virus, immunotherapy, invasive fungal diseases, infections, viral infections

## Abstract

Chimeric antigen receptor (CAR)-T immunotherapy has revolutionized the management of patients with relapsed/refractory B-cell hematological malignancies. There is emerging evidence that CAR-engineered cells—not only T cells, but also natural killers and macrophages—might have a crucial role in the treatment of autoimmune disorders and solid tumors. Moreover, given the burden of chronic infectious diseases, the mortality and morbidity of infections in immunocompromised individuals, and the development of multidrug-resistant pathogens, including bacteria, fungi, and mycobacteria, a need for novel and personalized therapeutics in this field is emerging. To this end, the development of CAR cells for the management of chronic infections has been reported. In this literature review, we summarize the ongoing clinical and pre-clinical data about CAR cell products in the field of infectious diseases. Currently, clinical studies on CAR immunotherapy for infections mainly concern human immunodeficiency virus infection treatment, and data regarding other infections largely originate from preclinical in vitro and in vivo models. In the era of personalized medicine, effective and safe therapies for the management of chronic infections and infectious complications in immunocompromised patients are crucial.

## 1. Introduction

Chimeric antigen receptor (CAR) immunotherapy, including engineered immune cells such as T cells (CAR-T), natural killer (NK) cells (CAR-NK), and macrophages engineered to express synthetic receptors (fusion proteins), can target antigens expressed on the cell surface and eliminate malignant or infected cells [1,2]. The first CAR was developed by Eshhar’s group in 1989 [2]. A CAR typically includes the following components: a targeting domain, commonly an antibody fragment like a functional single-chain variable fragment (scFv), that can recognize a specific antigen, a hinge region, a transmembrane domain, and intracellular signaling domains such as CD3ζ and costimulatory molecules, such as CD28 [3]. Initially, first-generation CARs showed limited effectiveness due to poor persistence. Notably, the incorporation of costimulatory signals in second-generation CARs has significantly enhanced their function, resulting in clinical success in the management of hematological malignancies. To date, only second-generation CAR-T cells for the treatment of B-cell malignancies have been approved for use in clinical practice [3]. 

CAR-T cell immune therapeutics have made a significant impact in the treatment of patients with relapsed or refractory (R/R) lymphomas and B-cell acute lymphoblastic leukemia (B-ALL) [4]. Currently, four CAR-T cell products—axicabtagene ciloleucel, brexucabtagene autoleucel, lisocabtagene maraleucel, and tisagenlecleucel—are approved for R/R B-cell hematological malignancies, while two others have been authorized by the Food and Drug Administration for R/R multiple myeloma: isocabtagene maraleucel and idecabtagene vicleucel [5]. Furthermore, real-world clinical experience supports both the efficacy and safety of CAR-T cell products, even though some patients may develop post-infusion complications [6,7]. These include early toxicities, such as cytokine release syndrome (CRS) and immune effector cell-associated neurotoxicity syndrome (ICANS), as well as hematological toxicities such as cytopenias—immune effector cell-associated hematotoxicity (ICAHT) [8,9]. Other complications described include infections, neurocognitive dysfunction, and thromboembolic events occurring both early and long-term after infusion [10,11,12]. 

Beyond hematological malignancies, CAR-T therapy is also under investigation for the treatment of autoimmune diseases, such as systemic lupus erythematosus and rheumatoid arthritis, and various solid tumors [13,14]. Furthermore, given the burden of chronic infectious diseases, such as acquired immunodeficiency syndrome (AIDS), the severity of viral and fungal infections in immunocompromised populations, and the emergence of multidrug-resistant pathogens, including bacteria, fungi, and mycobacteria, there is an unmet need for the development of novel and personalized therapeutics in this field [15,16,17]. To this end, the development of CAR immune products for the management of chronic infections might be beneficial [18]. To date, clinical studies on CAR immunotherapy for infections have mainly focused on human immunodeficiency virus (HIV) infection management, while data concerning other infections mainly derive from preclinical in vitro and in vivo models [19]. In this literature review, we aim to summarize the current clinical and pre-clinical data about CAR cell therapeutics in the field of infectious disease management. In the era of precision medicine, the development of safe and effective alternative therapies for both chronic infections and infectious complications experienced by vulnerable populations is of paramount importance.

## 2. HIV Infection

HIV infection, a major public health issue worldwide, results in chronic suppression of the immune system due to the depletion of CD4+ T lymphocytes. HIV-infected individuals experience an increased risk of opportunistic infections, secondary malignancies, and cardiovascular disease, while long-term treatment with daily antiretroviral therapy (ART) is essential for the suppression of HIV plasma viremia [20]. Thus, various research approaches, both pre-clinical and clinical, have been developed for the management of HIV infection with CAR-T cells, given the necessity of lifelong ART and the complications experienced by these patients [21]. The first studies published in the field concerned the development of first-generation genetically modified CAR-T products, which were characterized by an extracellular domain of CD4+ fused with the intracellular domain of the CD3ζ chain (CD4ζ-CAR), targeting infected cells by binding to HIV glycoprotein 120 (gp120) envelope protein. Early pre-clinical data demonstrated that CAR-T cells directed against CD4 can eliminate HIV-infected cells in vitro [22,23]. Moreover, second- and third-generation CARs, which include costimulatory domains, such as CD28 and 4-1BB, can enhance persistence and cytotoxic ability, as has been shown in pre-clinical models [24,25]. 

Mitsuyasu et al., in their phase II clinical trial, examined the efficacy of CAR-T cell immunotherapy in 24 HIV-positive patients [26]. Eleven patients received autologous CD4+ and CD8+ CAR T-cells containing the CD4ζ gene combined with interleukin-2 (IL-2) administration, while the rest of the study participants were treated with CAR-T cells alone. A greater than 0.5 log mean decrease in HIV ribonucleic acid (RNA), detected in rectal tissue, was observed for at least 14 days after the CAR-T infusion. Additionally, it was reported that in patients who received IL-2 along with CAR-T cell immunotherapy, CD4+ counts were substantially similar 8 weeks post-infusion compared to baseline levels (*p* = 0.10). Nevertheless, the mean change in HIV RNA or blood proviral deoxyribonucleic acid (DNA) levels was not significant in either of the two groups (CAR-T+IL-2).

In the phase I study of Walker and colleagues, patients with HIV received CAR-T cell infusion with CD4+ alone or combined with CD8+ modified T cells from identical twin donors, while multiple infusions were administered [27]. Sustained CAR-T cell survival in circulation, for at least 1 year, was achieved in patients who received both CD4+ and CD8+ CAR-T cell infusion. Moreover, the presence of modified cells in lymphoid organs, as assessed by biopsy, was lower or equivalent to that in circulation. CAR-T cell therapy was reported as safe without any significant adverse effects. In the phase II study of Deeks et al., 40 patients were included: 20 were treated with autologous gene-modified CD4+, CD8+ CAR T-cells, containing the CD4ζ gene, and 20 with unmodified T cells [28]. In all patients, CAR-T cell immunotherapy was combined with highly active ART administration. In both groups (gene-modified/unmodified T cell recipients), CD4+ T cells increased post-infusion. Furthermore, no significant differences in HIV reservoirs after the infusion were observed between the two groups. However, in patients who received gene-modified T cells, a substantial decrease in quantitative HIV coculture and rectal biopsy HIV DNA was reported. Overall, in the above-described trials, the long-term persistence of CAR-T cells in the circulation and the safety of this therapeutic approach were established [19,29]. Nevertheless, the main issue that arises concerns the lack of impact of anti-HIV CAR-T cell infusion on viral load, and consequently, it is difficult to consider its use as therapy for HIV infection.

Infusion of broadly neutralizing antibodies (BNAbs), targeting the CD4+ binding site on the HIV envelope protein, has been found to effectively reduce circulating viral load in several clinical trials [30,31,32]. BNAbs CAR-T cells are genetically modified T cells expressing CAR based on BNAbs [33]. Specifically, these T cells can recognize and kill virus-infected cells by targeting conserved viral regions, combining the precision of BNAbs with the cytotoxic power of T cells, serving as potential agents of antiviral therapy. In various preclinical studies, BNAb-derived HIV-1–specific CAR T cells have been found to suppress viral replication in animal models [34,35]. Based on the encouraging pre-clinical results, Liu et al. were among the first to investigate the safety and efficacy of BNAb-derived CAR-T cells in individuals living with HIV [36]. In this study, 15 patients were included, while in 6 of them, antiretroviral therapy was interrupted before the CAR-T infusion. CAR-T cell immunotherapy was reported as well-tolerated and safe. Additionally, a statistically significant decrease in HIV RNA levels was observed post-infusion, and, regarding the six patients who discontinued HAART, the median time to the viremia rebound was 5.3 weeks. 

**Figure 1:**
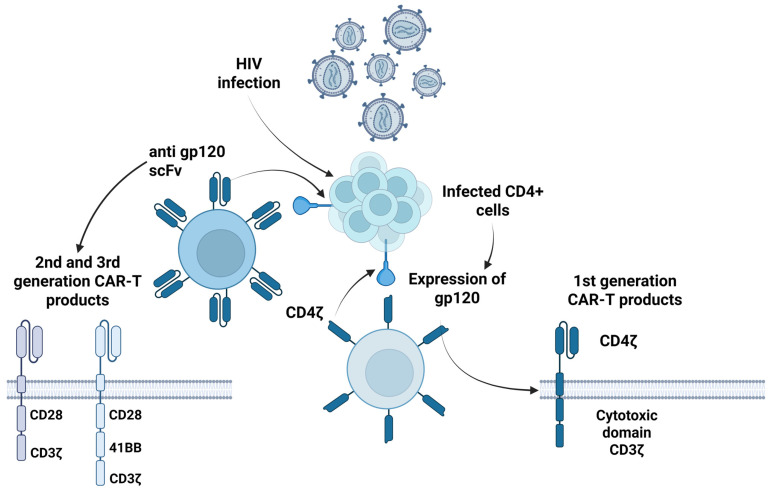
Mechanism of anti-HIV CAR-T cell immunotherapy targeting infected CD4+ cells expressing gp120. HIV infection results in expression of the envelope glycoprotein gp120 on the surface of infected CD4+ T cells. First-generation CAR-T cells are engineered to express a CD4ζ receptor, consisting of an extracellular CD4 domain combined with the CD3ζ intracellular cytotoxic domain, leading to recognition and elimination of gp120^+^ cells. In second- and third-generation CAR-T immune products, additional costimulatory domains, such as CD28 and 4-1BB, are incorporated in combination with the CD3ζ cytotoxic domain. Anti-gp120 scFvs have also been used for this aim, to enhance both T cell activation and cytotoxic function against HIV-infected cells. (Created in BioRender. Evangelidis, P. (2025) https://BioRender.com/tkidb6f, accessed on 22 April 2025) gp120: glycoprotein 120; HIV: human immunodeficiency virus; scFv: single-chain variable fragment.

Recently, Mao and colleagues, in their study, examined the safety and efficacy, both in vitro and in HIV-1-infected individuals, of M10 cells: allogeneic CAR-T cells, recognizing Env, with endogenous BNAbs and a follicle-homing C-X-C chemokine receptor type 5 (CXCR5) [37]. In vitro, M10 cells were found to exhibit broad cytotoxic effects on HIV-infected cells, while neutralizing cell-free viruses and B-cell follicle homing. M10 cells were administered in two infusions, with an interval of 30 days, in 18 patients, and each M10 cell infusion was followed by chidamide stimulations to activate the HIV-1 viral reservoir. In 74.3% of CAR-T cell recipients, a significant viral rebound was observed after an initial decrease in viral load, while the average decrease in viral load was reported at 67.1%. Additionally, in 10/18 patients, persistently reduced cell-associated HIV-1 RNA levels (average decrease of 1.15 log10) were reported during the follow-up period of 150 days. A selective pressure on the latent viral reservoir by M10 CAR-T cells was also found. Moreover, significant treatment-related adverse events were not reported. In Table 1, an overview of the published clinical trials examining the safety and efficacy of CAR-T cell immunotherapy against HIV is presented. Additionally, in Figure 1, the mechanisms of action of these CAR-T products are illustrated. Currently, several ongoing trials are examining the safety and efficacy of this approach in people living with HIV (Table 2).

## 3. EBV and CMV Infections (EBV: Clinical Trial)

Epstein–Barr virus (EBV) is the cause of infectious mononucleosis, a systemic infection that is self-limiting in immunocompetent individuals. However, in immunocompromised patients, severe and chronic EBV infections can be observed, and it has also been associated with the development of solid tumors, including nasopharyngeal carcinomas, and hematological malignancies, such as Burkitt’s lymphomas [43]. Latent membrane protein 1 (LMP1) is a protein in EBV’s cell membrane, crucial for viral reproduction [44]. Nevertheless, EBV presents distinct latency programs (latency types 1, 2, and 3), each characterized by specific latency gene expression patterns. Interestingly, LMP1 is absent in latency type 1, which has implications for the development of universally expressed EBV targets for CAR-T cell products. When EBV is latent, gene expression of the virus is limited, and thus cannot be recognized by the immune system [45]. Given that cytotoxic T and NK cells play a substantial role in the “fight” against acute EBV infection, and the fact that chronic and persistent infections are mainly observed in individuals with immunodeficiencies, CAR cell therapies against EBV have been examined in various pre-clinical studies [46].

Tang et al., in their in vitro study, developed second-generation CAR-T cells against EBV containing an anti-LMP1 scFv, a CD28 signaling domain, and the CD3ζ cytotoxic domain [47]. In vitro, CAR-T cells exhibited cytolytic properties against EBV-positive nasopharyngeal carcinoma cells, in which LMP1 was overexpressed while producing interferon-γ (IFN-γ) and interleukin-2 (IL-2). In vivo, in an animal model, CAR-T cell infusion resulted in a significant decrease in tumor growth. 

Post-transplant lymphoproliferative disease (PTLD) is a chronic EBV-associated disorder observed in patients who undergo allogeneic hematopoietic cell transplantation (allo-HCT) or solid organ transplantation [48]. Despite the therapeutic advances that have been made in this field, the morbidity that these patients experience remains high [49]. Dragon and colleagues in their in vivo study developed T cells with a CAR based on the monoclonal antibody TÜ165, which recognizes a nuclear antigen of EBV (EBNA) (3C-derived peptide in HLA-B*35) [50]. Additionally, T cells capable of universal cytokine-mediated killing (TRUCKs) of PTLD cells were produced via IL-12 signaling. Co-cultivation of CAR-T and TRUCK cells with EBV (+) PTLD cells resulted in an increase in CAR-T activation markers, such as CD137 and CD25; in proinflammatory cytokines, including IFN-γ and tumor necrosis factor-α (TNF-α); and apoptosis-associated molecules (granzyme B and perforin), while IL-12 resulted in NK cell and monocyte recruitment. 

Glycoprotein 350 (gp350) on the surface of EBV cells, a common target of naturally occurring neutralizing antibodies, is a promising target for the management of EBV-associated disorders, such as PTLD [51]. Slabik et al., in their experimental study, investigated the impact of CD8+ CAR-T cell infusion [52]. In 75% of mice, EBV spread was controlled or reduced, exhibiting a lack of tumor development and reduced inflammation. In addition, preclinical CAR-T cell products against gp350 have been investigated for EBV (+) lymphomas [53]. Data from clinical trials in humans regarding the safety and efficacy of CAR-T cell products against EBV-associated chronic infections are lacking, and more research is essential in this field [54]. Several clinical trials in this field, especially for EBV-associated malignancies and CD30 (+) Hodgkin lymphoma, are ongoing [55].

Cytomegalovirus (CMV) can cause severe infections in immunocompromised patients, such as pneumonia, retinitis, and colitis, while at the same time predisposing them to the development of an immunosuppressed state, and consequently, secondary opportunistic infections [56]. Interestingly, CMV replication has been associated with the development of acute graft-versus-host disease in allo-HCT recipients [57]. Despite the introduction of novel antiviral therapies in everyday clinical practice, toxicities and resistance might be observed [58]. Adoptive cell therapy with the infusion of T cells from healthy latently CMV-infected donors to allo-HCT recipients with post-transplantation refractory CMV infection has been examined with positive results in clinical practice. In contrast, barriers, such as the limited number of available donors, still exist [58,59,60,61]. 

Based on the morbidity burden that immunocompromised patients with severe/refractory infections experience, several research groups have examined, in preclinical models, the efficacy of CAR-T cell therapeutics against CMV [62]. Glycoprotein B, on the cell surface, is the most common target of the developed CAR-Ts, and it is essential for virus spread [63]. Full and colleagues were the first to develop CAR-T cells against CMV with an scFv against CMV glycoprotein, a CD28 domain, and a cytotoxic CD3ζ region [64]. However, resistance in the elimination of CMV-infected cells was observed, which might be due to the release of antiapoptotic molecules by CMV viral cells [65]. Olbrich et al. examined the efficacy of CAR-T cells targeting CMV glycoprotein B using high-affinity antibody-derived scFvs and 4-1BB or CD28 costimulatory domains [66]. It was reported that in vitro, 4-1BB-based anti-glycoprotein B CAR-T cells showed superior activation and cytotoxicity compared to CD28-based ones, while in vivo testing performed in humanized mice demonstrated effective viral control in most cases. Ali and colleagues developed eight different CMV-specific CARs using BNAbs sequences and successfully expressed them in primary CD8+ T cells [67]. Among them, the CAR-T based on antibody 21E9 consistently demonstrated the strongest antiviral activity against CMV across multiple functional assays. 

## 4. Hepatitis B and C Infections 

Infections from the hepatitis B virus (HBV) and hepatitis C virus (HCV) affect millions of individuals worldwide [68,69]. In particular, HBV chronic infections are associated with increased morbidity and mortality, mainly attributed to the lack of effective and targeted therapeutics. Thus, HBV is considered an optimal target of CAR-T cell therapeutics. In most of the published preclinical studies, CAR-T cells with scFvs targeting hepatitis B surface antigen (HBsAg) in the cell membranes of HBV-infected hepatocytes have been used. It must be highlighted that CAR-Ts against both small surface (S) and large surface (L) proteins as components of HBsAg have been developed. The in vitro study by Bohne and colleagues aimed to eliminate HBV covalently closed circular DNA (cccDNA) by developing CAR-T cells to target infected hepatocytes expressing HbsAg [70]. Researchers engineered CAR-Ts using scFvs against HBsAg S or L proteins, fused with CD3ζ and CD28 signaling domains. These modified CAR-T cells successfully recognized and lysed HBV-infected, cccDNA-positive hepatocytes, while a release of cytokines such as IFN-γ and IL-2 was observed [70]. 

Kruse et al. developed a novel HBsAg-specific CAR-T cell product and examined its effectiveness against HBV infection. In vitro, the CAR-T cells recognized HBV-positive cells and HBsAg proteins, but cytotoxic activity was not observed. In a humanized mouse model with HBV infection, the CAR-T cells significantly reduced plasma HBsAg and HBV-DNA levels and the number of HBV-infected hepatocytes without causing liver damage [71]. In a study by Klopp et al., mechanisms for reducing HBV-specific T-cell therapy-associated risks for liver toxicity and CRS were evaluated [72]. T cells were engineered to express HBV-specific CARs or T-cell receptors (TCRs) along with the addition of either inducible caspase 9 (iC9) or herpes simplex virus thymidine kinase (HSV-TK) as safety “switches”. Activation of these “switches” rapidly inhibited cytotoxic activity and efficiently depleted T cells in vivo, particularly in the liver, significantly limiting toxicity. Nevertheless, a reduced antiviral effectiveness was reported, as most remaining T cells evolved into non-functional cells. 

Festag and colleagues developed second-generation CAR-T cells targeting the S protein of HBV [73]. In an immunocompetent mouse model, human-derived CAR-T cells co-expressing safeguard truncated epidermal growth factor receptor (EGFRt) protein were infused, triggering immune responses that limited the survival of the engineered T cells. To resolve this issue, induction of immune tolerance was performed with total body irradiation and transfer of signaling-deficient CAR-T cells, allowing therapeutic CAR-T cells to persist after immune recovery. Similarly, Guo et al., in their study, investigated the efficacy of HBV-specific CAR T cells with 2H5-A14 antibody targeting the pre-S1 region of the HBV envelope protein. These A14 CAR T cells effectively eliminated HBV-infected hepatocytes and reduced viral markers to undetectable levels in humanized mice. Furthermore, an induction of antiviral cytokine production was observed, suggesting a novel potential curative therapy against HBV infection [74]. The recently published study by Wang et al. focused on the development of novel CARs using scFvs (MA18/7 and G12) against HBV envelope proteins [75]. The CARs, particularly those based on G12, effectively eliminated HBV infection and reduced HBsAg secretion by restraining viral envelope proteins in the endoplasmic reticulum. G12-scFv-crystallizable fragments (Fc) and G12-CAR-Fc formats showed significant antiviral effects in HBV mouse models, reducing serum levels of HBsAg. More data regarding the safety of CAR-T immunotherapy is essential, as, to date, clinical studies in this field have not been performed.

Data regarding CAR immunotherapy for HCV infection are limited. The in vivo study by Sautto et al. introduced the first CAR-T cells for the management of HCV infection, targeting the E2 glycoprotein on the virus cell surface using scFvs from BNAbs [76]. The engineered T cells specifically recognized and killed cells expressing E2 glycoprotein, including infected hepatocytes. Moreover, secretion of antiviral and proinflammatory cytokines, such as IFN-γ, IL-2, and TNF-α, by CAR-T cells was observed. However, given the significant therapeutic advances made in the management of HCV infection in recent years, the role of such a therapeutic approach is potentially limited [77]. 

## 5. SARS-CoV-2 Infection (Clinical Trials)

The coronavirus disease (COVID-19) pandemic caused by the SARS-CoV-2 has been identified as a major cause of acute respiratory disease, resulting in millions of deaths worldwide over the last few years [78,79]. Virus-specific T-cell immunotherapy has been found effective in COVID-19 management [80,81,82]. Moreover, in several preclinical studies, the efficacy of CAR immune therapeutics has been evaluated. A study by Guo et al. presents an approach for SARS-CoV-2 infection management with CAR-T cells engineered to recognize the virus’s receptor-binding domain (RBD), leading to T cell activation and production of IFN-γ, granzyme B, and perforin [83]. The CAR-Ts showed strong in vitro elimination of RBD- or spike protein (S1)-expressing target cells, with cytotoxicity primarily mediated through the granzyme B/perforin pathway. Furthermore, Dogan and colleagues developed CD8 T cells with angiotensin-converting enzyme 2 (ACE2)-based CARs and engineered a bispecific T cell engager (ACE2-Bite) to selectively kill S1-expressing cells and neutralize the virus [84]. ACE2-Bite showed strong cytotoxicity and neutralization against SARS-CoV-2 wild-type, Delta, and Omicron variants, with higher affinity for Delta and Omicron. 

Beyond T cell-based CAR-immunotherapy, macrophages and NK cells have been used in preclinical studies [85]. Tuyet Ma et al. developed S309-CAR-NK cells using the scFv domain from the BNAb S309, targeting a conserved region of the SARS-CoV-2 S1 protein [86]. S309-CAR-NK cells specifically bound to and eliminated cells expressing S1 protein, while demonstrating an ability to kill these cells in vitro. Additionally, in the experimental study by Fu et al., human macrophages with CARs to enhance their ability to clear SARS-CoV-2 were developed [87]. While CARs with different intracellular domains showed varying abilities to trigger phagocytosis and killing, all mediated similar viral clearance in vitro. Notably, CARMERTK macrophages reduced the viral load without inducing the production of proinflammatory cytokines, suggesting a potential "immunologically silent" therapy for severe COVID-19. However, more data from clinical trials are essential to establish CAR immunotherapy for COVID-19. There is an open-label, randomized, multicenter phase I/II clinical trial (NCT04324996) evaluating the therapy with CAR-NK cells in 90 COVID-19 patients across mild, severe, and critical infection cases [88]. Modified NK cells, expressing NKG2D CAR, an activating receptor of these cell types, can recognize SARS-CoV-2-infected cells and ACE2, while targeting the virus directly. To improve NK cell persistence and safety, the cells were modified to secrete an interleukin-15 superagonist and a granulocyte-macrophage colony-stimulating factor-neutralizing scFv. These off-the-shelf NKG2D-ACE2 CAR-NK cells will be derived from cord blood, designed to both eliminate virus-infected cells and prevent CRS.

## 6. Invasive Fungal Diseases

Invasive fungal diseases (IFDs) are considered a major cause of mortality and morbidity in immunocompromised patients, especially those with hematological malignancies [89,90]. Importantly, patients who undergo allo-HCT are at a higher risk of IFDs due to several causes, such as treatment with immunosuppressive agents for graft-versus-host disease, high-dose corticosteroids, and haploidentical/unrelated allogeneic HCT [91]. The majority of IFD cases are attributed to *Aspergillus* spp. and *Candida* spp. [92,93]. Specifically, *Aspergillus* spp. infections in immunocompromised individuals can lead to the development of severe pulmonary disease, specifically, invasive pulmonary aspergillosis (IPA). The increased mortality due to IFDs in vulnerable populations, the emergence of antifungal-resistant isolates, and the side effects of current antifungal drugs, highlight that CAR-based immunotherapy might be beneficial in this field [94]. 

Kumaresan et al., in their experimental study, developed T cells with a CAR, based on the Dectin-1 receptor (D-CAR), targeting fungal β-glucan [95]. Specifically, genetically modified D-CAR-T cells were expanded on several artificial activating cells. Interestingly, the modified T cells effectively caused damage to and inhibited *Aspergillus* hyphae growth both in vitro and in vivo, even after the administration of corticosteroids. Thus, in this study, a promising strategy to enhance antifungal immunity was demonstrated for the first time. As *Aspergillus fumigatus* is considered an important cause of IFDs in patients with hematological malignancies and other immunocompromised individuals [96], Seif and colleagues, in a preclinical model, showed that *Aspergillus fumigatus*-specific CAR-T cells (Af-CAR), with an AB90-E8 targeting domain recognizing an antigen in the cell wall of fungal hyphae, were developed [97]. Af-CAR-T cells showed strong antifungal activity, releasing perforin and granzyme B, and leading to macrophage activation. In a mouse model of invasive pulmonary aspergillosis, infusion of CD8+ Af-CAR-T cells resulted in a reduction in fungal burden and improved survival [98]. In the study by Bauser et al., a method for the generation of CAR-engineered NK cells was described using the non-viral Sleeping Beauty transposon system [99]. NK-92 cells were transfected with minicircle DNA encoding the hyperactive SB100X transposase and a CAR transposon, followed by magnetic enrichment and subsequent expansion. In functional assays, antigen-specific activation of CAR-NK cells was confirmed upon co-culture with *Aspergillus fumigatus*, as demonstrated by the secretion of IFN-γ. 

**Figure 2:**
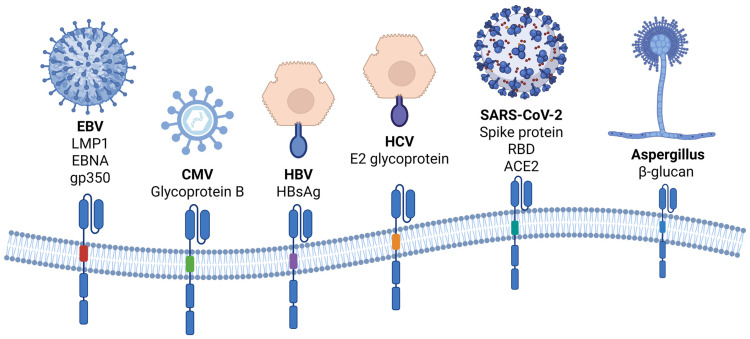
An overview of CAR cells engineered against viral and fungal pathogens. Various antigens of pathogens have been used as targets of CARs. (Created in BioRender. Evangelidis, P. (2025) https://BioRender.com/3n28kf2, accessed on 22 April 2025) ACE2: angiotensin-converting enzyme 2; CAR: chimeric antigen receptor; CMV: cytomegalovirus; EBNA: Epstein–Barr virus nuclear antigen; EBV: Epstein–Barr virus; gp350: glycoprotein 350; HBsAg: hepatitis B surface antigen; HBV: hepatitis B virus; HCV: hepatitis C virus; LMP1: latent membrane protein 1; RBD: receptor-binding domain.

*Cryptococcus* species can cause severe infections in immunocompromised patients, such as those living with HIV [100]. The sugar-based capsule of *Cryptococcus* is crucial for infection pathogenesis [97]. Aparecido da Silva et al., in order to target the sugar-based capsule, developed CD8+ T cells with a glucuronoxylomannan (GXM)-specific CAR (GXMR-CAR) which had the ability to bind to the capsule’s GXM [101]. It must be underlined that GXMR-CAR-T cells exhibited specific GXM recognition capability while secreting granzyme and IFN-γ post-CAR-T cell activation. Moreover, GXMR-CAR-T cells were found to bind to *Cryptococcus neoformans*, reducing the number of giant cells in infected lung tissues. Nevertheless, data regarding CAR-T cell immunotherapy of IFDs are, to date, based on preclinical in vitro models, and thus rigorous investigation in animal in vivo studies is of paramount importance prior to its introduction in clinical practice. In Figure 2, the antigen targets of CAR cells described above are presented.

## 7. Conclusions and Future Perspectives 

During the last few years, CAR-based immunotherapies have been investigated, mainly on preclinical models, for the management of chronic infectious diseases, with promising results. Regarding HIV, early phase I/II clinical trials have shown the safety and persistence of CAR-T cells post-infusion, especially when combined with the integration of BNAbs. Additionally, encouraging results have arisen from preclinical studies concerning other persistent/refractory viral infections, such as EBV, CMV, HBV, and SARS-CoV-2. While first-, second-, and third-generation CAR-T cell products have been investigated for the management of infectious diseases, they present differences in their structure, persistence, exhaustion susceptibility, and immune escape risk, as summarized in Table 3.

However, important obstacles limit the further application of this approach in everyday clinical practice. Challenges include the limited in vivo persistence of CAR-T cells, as has been shown in some trials in HIV; the risk of CRS development; the complexity of manufacturing personalized cell products; and increased costs. Furthermore, while in preclinical studies, encouraging results have been found, large-scale clinical trials are essential for the evaluation of the safety, efficacy, and long-term outcomes of such an approach. Multicenter collaboration for the organization of clinical trials in this field and multidisciplinary teams are crucial to achieve this. Additionally, toxicities, such as CRS and ICANS, might also manifest in the post-infusion period, like those after CAR-T immunotherapy for hematological malignancies. Thus, early recognition, diagnosis, and grading are considered important.

## Figures and Tables

**Table 1:** An overview of the published clinical trials examining the safety and efficacy of CAR-T cell immunotherapy against HIV.

First Author, Year of Publication, Reference	Number of Participants	Age of Participants (Years)	Phase	CAR-T Generation	Type of CAR-T	Significant Findings
Mitsuyasu, 2000, [26]	24	Mean (range): 40 (29–50)	II	1st	Autologous CD4+, CD8+ CAR T-cells containing CD4ζ genes with or without IL-2 administration	Mean change in levels of HIV RNA or blood proviral DNA was not significant in either of the two groups (CAR-T+IL-2)
Walker, 2000, [27]	30	NS	I	1st	Single or multiple infusions of CD4+ and CD8+1 CAR-T cells from identical twin donors with CD4/CD3-z gene	-Sustained CAR-T cell survival in circulation, for at least 1 year, was achieved in patients who received both CD4+ and CD8+. -The presence of modified cells in lymphoid organs was lower or equivalent to that in circulation. -CAR-T cell therapy was safe.
Deeks, 2002, [28]	401 (20 modified and 20 unmodified T cells)	Mean age (range): -Gene modified group: 39 (28, 54) -Unmodified: 43 (28, 59)	II	1st	Autologous CD4+, CD8+ CAR T-cells containing CD4ζ gene combined with HAART administration	-In both groups (gene-modified/unmodified), CD4+ T cells were increased post-infusion. -No significant differences in HIV reservoirs. -In patients who received gene-modified T cells, a significant decrease in two viral reservoirs was reported (quantitative HIV coculture, rectal biopsy HIV DNA).
Liu, 2021, [36]	15	Median (range): 31 (26–47)	I	3rd	CAR-T cells2 with endogenous BNAbs	-CAR-T cells were well-tolerated and safe. -6 patients discontinued HAART: the median time to the viremia rebound was 5.3 weeks. -Statistically significant decrease in HIV RNA levels post-infusion.
Mao, 2024, [37]	18	Median (range): 31 (18–57)	I	2nd	Allogeneic CAR-T cells, recognizing Env, with endogenous BNAbs and a follicle-homing receptor CXCR5 (M10 cells)3	-In vitro, M10 cells were found to exhibit broad cytotoxic effects on HIV-infected cells, while neutralizing cell-free viruses and B-cell follicle homing. -74.3% of CAR-T cell recipients exhibited a significant viral rebound (after an initial decrease) -Average 67.1% decrease in viral load. -In 10/18 patients, persistently reduced cell-associated HIV-1 RNA levels (average decrease of 1.15 log10) were reported during the follow-up (150 days). -No significant treatment-related adverse events.

BNAbs: broadly neutralizing antibodies; CAR-T: chimeric antigen receptor-T; CXCR5: C-X-C motif chemokine receptor 5; Env: envelope; HAART: highly active antiretroviral therapy; HIV: human immunodeficiency virus; IL-2: interleukin 2; NS: not stated; RNA: ribonucleic acid. (1) In 17 patients, both CD4+ and CD8+ CAR-T cells were infused. (2) In 6 patients, antiretroviral therapy was interrupted. (3) M10 cells were administered in two infusions, with an interval of 30 days. Each M10 cell infusion was followed by stimulations with chidamide to activate the HIV-1 viral reservoir.

**Table 2:** An overview of the published clinical trials examining the safety and efficacy of CAR-T cell immunotherapy against HIV.

Clinical Trial Registration Number	Country	Title	Phase, Status
NCT06880380 [38]	China	The Efficacy and Safety Study of CAR-T Cells for Functional Cure in HIV-1/AIDS Patients (HIV-CAR-T)	I, Not yet recruiting
NCT03240328 [39]	China	The Effect of CAR-T Cell Therapy on the Reconstitution of HIV-specific Immune Function	I, Recruiting
NCT06252402 [40]	United States	CMV-specific HIV-CAR T Cells as Immunotherapy for HIV/AIDS	Early I, Recruiting
NCT04863066 [41]	China	Third-Generation CAR-T-cell Therapy in Individuals With HIV-1 Infection (TCTIWHI)	I, Unknown status
NCT04648046 [42]	United states	CAR-T Cells for HIV Infection	I/II, Recruiting
NCT03617198	United States	CD4 CAR+ ZFN-modified T Cells in HIV Therapy	I, Active/Not Recruiting

AIDS: acquired immunodeficiency syndrome, CAR-T: chimeric antigen receptor-t, CMV: cytomegalovirus, HIV: human immunodeficiency virus, ZFN: zinc-finger nuclease.

**Table 3:** Summary of the significant differences between 1st, 2nd, and 3rd generation CAR-T cell products for the management of infectious diseases.

	1st Generation CAR	2nd Generation CAR	3rd Generation CAR
Structure-costimulatory domains	None (only CD3ζ)	One (such as CD28 or 4-1BB)	Two (such as CD28 and 4-1BB)
Persistence	Low	Improved persistence due to costimulation	Enhanced persistence
Exhaustion susceptibility	High–early exhaustion	Moderate–costimulation delays exhaustion	Lower–dual signaling mitigates exhaustion
Immune escape risk	Higher–insufficient persistence	Moderate–improved antigen clearance	Lower–sustained immune pressure on pathogens

CAR: chimeric antigen receptor.

## Abbreviations

The following abbreviations are used in this manuscript:

ACE2	angiotensin-converting enzyme 2
Af-CAR	*Aspergillus fumigatus*-specific chimeric antigen receptor
AIDS	acquired immunodeficiency syndrome
allo-HCT	allogeneic hematopoietic cell transplantation
ART	antiretroviral therapy
BNAb	broadly neutralizing antibody
CAR	chimeric antigen receptor
cccDNA	covalently closed circular DNA
CMV	cytomegalovirus
COVID-19	Coronavirus disease-19
CRS	cytokine release syndrome
CXCR5	C-X-C chemokine receptor type 5
D-CAR	chimeric antigen receptor targeting Dectin-1 receptor
DNA	deoxyribonucleic acid
E2	E2 glycoprotein
EBNA	Epstein–Barr nuclear antigen
EBV	Epstein–Barr virus
EGFRt	truncated epidermal growth factor receptor
Fc	crystallizable fragment
gp350	glycoprotein 350
gp120	glycoprotein 120
GXM	glucuronoxylomannan
GXMR-CAR	glucuronoxylomannan-specific chimeric antigen receptor
HBsAg	hepatitis B surface antigen
HBV	hepatitis B virus
HCV	hepatitis C virus
HIV	human immunodeficiency virus
HSV-TK	herpes simplex virus thymidine kinase
iC9	inducible caspase 9
IFDs	invasive fungal diseases
IFN-γ	interferon-γ
IL-12	interleukin-12
IL-2	interleukin-2
IPA	invasive pulmonary aspergillosis
L	large surface proteins
LMP1	latent membrane protein 1
MERTK	Mer tyrosine kinase
NK	natural killer
PTLD	post-transplant lymphoproliferative disease
RBD	receptor-binding domain
RNA	ribonucleic acid
S	small surface proteins
S1	spike protein
scFv	functional single-chain variable fragment
TCR	T-cell receptor
TNF-a	tumor necrosis factor-α
TRUCKs	T cells capable of universal cytokine-mediated killing
